# Dopaminergic hypersensitivity of the opioid-responsive striatal–entopeduncular pathway in a rodent model of restless legs syndrome

**DOI:** 10.1038/s41386-026-02413-2

**Published:** 2026-04-17

**Authors:** Marta Valle-León, William Rea, César Quiroz, William N. Sánchez, Sandra Albornoz, Ana M. Rule, Ning-Sheng Cai, Francisco Ciruela, Christopher J. Earley, Sergi Ferré

**Affiliations:** 1 Integrative Neurobiology Section, National Institute on Drug Abuse, Intramural Research Program, National Institutes of Health, Baltimore, MD USA; 2 Center for Restless Legs Study, Department of Neurology, Johns Hopkins University, Baltimore, MD USA; 3 Department of Environmental Health and Engineering, Johns Hopkins Bloomberg School of Public Health, Baltimore, MD USA; 4 Department of Pathology and Experimental Therapeutics, School of Medicine and Health Sciences, Institute of Neurosciences, University of Barcelona, L′ Hospitalet de Llobregat, Spain

**Keywords:** Diseases of the nervous system, Neurotransmitters

## Abstract

We hypothesized that Restless Legs Syndrome (RLS) and the restlessness of opioid withdrawal share common neurobiological mechanisms based on the efficacy of μ-opioid receptor (MOR) agonists in RLS and the common RLS-like phenotype of patients with opioid withdrawal. We also hypothesized this involves an increased sensitivity of the striatal striosomal neurons that co-express MORs and dopamine D_1_ receptors (D_1_Rs) and release GABA in the internal segment of the globus pallidus (GPi). This hypothesis was tested in mice with diet-induced brain iron deficiency (BID), a rodent model of RLS. Fiber-photometry experiments were performed in mice with BID using a viral GABA biosensor injected in the entopeduncular nucleus (EPN), the GPi equivalent in rodents. EPN GABA release was measured after the systemic administration of the D_1_R agonist SKF81297 and the MOR agonist methadone. Locomotor activation and striatal mRNA expression of D_1_Rs, MORs and adenosine A_1_ receptors (A_1_Rs) were also analyzed. A minimal locomotor-activating dose of SKF81297 induced a significant EPN GABA release in mice with BID but not controls, while a maximal locomotor-activating dose of the MOR agonist methadone significantly reduced EPN GABA release in mice with BID after saline or SKF81297 administration. BID was associated with a significant reduction in the striatal expression of MORs and A_1_Rs. The results indicate that BID induces an increased dopaminergic sensitivity of the opioid-responsive striatal–EPN pathway, which might represent a pivotal pathogenetic mechanism of the restlessness of RLS and opioid withdrawal.

## Introduction

Restless legs syndrome (RLS) is a common neurological disorder characterized by an irresistible urge to move the legs (akathisia), triggered by rest, relieved by movement, and exhibiting a strong circadian pattern [1]. Patients consequently experience difficulty sitting, resting, or sleeping at night [1]. As a result, patients often experience an inability to sit, rest, or sleep at night [1]. In the 1950s, Nordlander showed that intravenous iron markedly improves RLS symptoms, even in non-anemic patients [2]. Subsequent studies identified altered brain iron homeostasis, with brain iron insufficiency emerging as a central factor in RLS pathophysiology [3], leading to development and validation of the brain iron deficiency (BID) rodent model of RLS [4].

In 1987, Akpinar reported dramatic improvement with levodopa [5], which later led establishing dopamine D_2_ receptor agonists as standard RLS therapy. Akpinar also proposed a combined dopaminergic and endogenous opioid dysfunction in RLS [5]. Evidence for opioid efficacy in RLS dates back to the 17th century [5, 6], and autopsy and PET studies support opioid involvement in RLS pathophysiology [7, 8]. Moreover, restlessness during opioid withdrawal often meets RLS diagnostic criteria [9, 10], suggesting shared neurobiological mechanisms involving opioid-responsive basal ganglia circuitry [11].

Given the efficacy of dopamine and μ-opioid receptor (MOR) agonists, interactions between the dopaminergic and opioid systems likely contribute to RLS expression [12], particularly within the striatum [13–15]. The striatum comprises matrix and striosomal compartments [16]. Striosomes, were initially defined as “*striatal islands of closely packed opioid receptors”* [17], which correspond to MORs predominantly expressed by striatal GABAergic efferent neurons in dendritic areas in apposition with dopamine terminals [15] and that primarily express dopamine receptors of the D_1_ subtype (D_1_Rs) [13, 14]. Striosomal MOR-D_1_R neurons differ from matrix D_1_R neurons in their outputs: matrix D_1_R neurons project to GABAergic neurons of the substantia nigra pars reticulata (SNpr) and the internal globus pallidus (GPi; entopeduncular nucleus, EPN, in rodents), which project to the thalamus. In contrast, striosomal MOR-D_1_R neurons project to glutamatergic GPi/EPN neurons that innervate the lateral habenula (lHb) [14, 18] (see Discussion).

Recent work showed that MOR activation in striosomal MOR-D_1_R neurons suppresses GABA release in the EPN, whereas opioid withdrawal enhances GABA release [14]. We therefore hypothesized that heightened sensitivity of this opioid-responsive striatal–EPN pathway could underlie restlessness shared by RLS and opioid withdrawal [11]. This hypothesis was tested in the BID mouse model of RLS [4] by assessing EPN GABA release induced by the dopamine D_1_R agonist SKF81297 (SKF) using in vivo fiber photometry. The contribution of striosomal versus matrix D_1_R neurons was inferred from responsiveness to the MOR agonist methadone. Locomotor responses to SKF were also compared between BID and control mice. Because BID enhanced SKF-induced EPN GABA release without increasing locomotion, additional experiments evaluated the effects of SKF, methadone, and their combination in naïve and reserpinized mice to assess presynaptic and postsynaptic MOR modulation of D_1_R-mediated locomotor activity. Finally, we examined BID effects on striatal mRNA expression of D_1_R, MOR, and the adenosine A_1_ receptor (A_1_R), a modulator of D_1_R signaling that is downregulated in BID [19–21].

## Materials and methods

### Animals, iron-deficient diet and reserpinization

Male and female C57BL/6 mice (Charles River Laboratories, Wilmington, MA) were used. Mice were group-housed (four per cage) under controlled conditions (12-h light/dark cycle) with ad libitum access to food and water. All procedures followed the National Institutes of Health Guide for the Care and Use of Laboratory Animals and were approved by the National Institute on Drug Abuse Intramural Program Animal Care and Use Committee (protocols 22-MTMD-21, 22-MTMD-23, 24-MTMD-14, and 25-MTMD-23). Locomotor and fiber-photometry experiments were conducted in ~9-week-old mice. Some locomotor studies were performed in reserpinized animals. Reserpine (Tocris Bio-Techne, Minneapolis, MN) was dissolved in a drop of glacial acetic acid, brought to volume with 5.5% glucose, and administered subcutaneously (5 mg/kg) 20 h before locomotor testing. This dose has been shown to induce marked striatal dopamine depletion (>95%) in mice [22]. For BID experiments, postweaning mice (3 weeks old) were randomly assigned to a control diet (48 ppm iron; TD.110846, Envigo, Indianapolis, IN, USA) or a low-iron diet (4 ppm iron; TD.09127, Envigo). Diets were maintained for six weeks before locomotor or fiber-photometry experiments and continued until study completion. Each animal participated in a single experiment and received only one diet and systemic treatment. Data from all experiments—including locomotor activity, in vivo EPN GABA release, and ex vivo assessments of striatal iron content and receptor mRNA—were pooled across male and female mice (~1:1), as preliminary analyses showed comparable results in both sexes.

### Assessment of brain iron deficiency

Dorsal striatum samples (~50 mg) were dissected, weighed, and stored at −80 °C until analysis. Samples were digested in 3 mL Optima™ grade nitric acid (HNO₃) using a MARS6 Xpress microwave system (CEM Corporation, Matthews, NC). A 500 μL aliquot of the digest was diluted to 10 mL in 2% HNO₃ and 0.5% HCl. Scandium (10 μg/L; CPI International, Santa Rosa, CA) was used as an internal standard, and accuracy was verified with Seronorm™ Trace Elements Whole Blood reference material (Sero AS, Blåsingstad, Norway). Total iron content was measured by inductively coupled plasma triple quadrupole mass spectrometry (ICP-QQQ-MS; 8900 ICP-MS/MS, Agilent Technologies) as previously described [23].

### Locomotor activity recording

Mice (9 weeks old) were used for locomotor experiments. In non-reserpinized animals, locomotor activity was recorded in 25 × 25 cm open-field chambers (ActiTrack software, Panlab-Harvard Apparatus, Barcelona, Spain) after 2 h habituation, immediately following i.p. administration of the D_1_R agonist SKF or vehicle, or 15 min after s.c. methadone (MOR agonist) or vehicle. Injection volume was 10 mL/kg in all experiments. The same protocol was applied to reserpinized mice, except that habituation was omitted due to reserpine-induced immobility preventing exploratory behavior before drug administration [22, 24]. Activity counts were recorded in 10-min bins, square root transformed to reduce right-skewness and variance differences [24], and averaged over 1 h for analysis.

### Fiber-photometry

After two weeks of dietary treatment, mice were anesthetized (ketamine/xylazine, 60–100/5–15 mg/kg, i.p.) and placed in a stereotaxic frame. An adeno-associated virus encoding the GABA biosensor iGABASnFR [25] (AAV2/1.hSynapsin1.iGABASnFR; 0.3 μL, 2.1 × 10¹³ GC/mL) was unilaterally injected into the EPN (AP − 1.3 mm, ML + 1.7 mm, DV − 4.5 mm from bregma) at 0.03 μL/min. The needle remained in place for 10 min post-infusion. Three weeks later, a chronic fiber-optic implant (200 µm core, 0.50 NA; Thorlabs, Newton, NJ) was placed at the same coordinates, with depth verified by real-time fluorescence, and secured with screws and dental cement. Recordings were performed using a dual-wavelength fiber-photometry system (405 nm isosbestic, 465 nm biosensor; emission 500–550 nm; Doric Lenses, Québec, Canada). Patch cords were photobleached for 2 h before use. Mice were placed in rotating chambers, tethered via a rotary joint, allowed to stabilize (first 40 min excluded), and a 10-min baseline was recorded before systemic administration of muscimol, SKF81297 hydrobromide, or methadone (saline vehicle). Fluorescence was continuously recorded post-injection. Signals were acquired with DoricStudio, analyzed using pMAT v1.223 and custom MATLAB R2023b scripts. The demodulated 465 nm signal, reflecting GABA biosensor activity, was normalized to the 405 nm signal to correct for motion artifacts and nonspecific fluctuations, expressed as ΔF/F, and plotted over time using GraphPad Prism v10.0. After experiments, mice were deeply anesthetized, perfused with PBS followed by 4% formaldehyde, and brains were processed as described [26]. Probe placement and iGABASnFR expression in the EPN were verified from 40-µm coronal brain sections using a Typhoon multimode laser scanner (GE Life Sciences, Piscataway, NJ).

### RT-qPCR

Quantitative RT-PCR was performed using a LightCycler® 480 II (Roche Diagnostics, Rotkreuz, Switzerland) to assess gene expression in striatal tissue from mice on ID or control diets. After dietary treatment, the striatum (~20 mg) was dissected and total RNA extracted using the RNeasy Mini Kit (Qiagen, Redwood City, CA). RNA was reverse transcribed into cDNA (Takara Bio, Madison, WI). RT-qPCR reactions (20 μL) contained 10 μL Luna® Universal qPCR Master Mix (New England Biolabs), 0.5 μL each forward and reverse primers (10 μM), 2 μL cDNA, and RNase-free water. Samples were run in triplicate, and mean Ct values were analyzed. Gene expression was normalized to *Gapdh* using the ΔΔCt method. *Gapdh* was chosen as housekeeping control gene because its mouse brain expression was previously shown to be insensitive to diet and the daily light-dark cycle [27]. Ct was defined as the cycle at which fluorescence exceeded a predefined threshold indicating the first detectable level of amplification. Primers were designed using the NCBI Primer-BLAST tool. Sequences were: 5’-gtctcccagatcgggcatttg-3’ (D_1_R forward), 5′-agtcacttttcggggatgct -3′ (D_1_R reverse), 5′- atcctctgagggagcgactg -3′ (A_1_R forward), 5′- gaggccagcacccaagg -3′ (A_1_R reverse), 5′- acaaaatacaggcaggggtcc -3′ (MOR forward), 5′- gacagcatgcggacactctt -3′ (MOR reverse), 5′-atggtgaaggtcggtgtga-3′ (GADPH forward), and 5′-aatctccactttgccactgc-3′ (GADPH reverse).

### Statistical analysis

Locomotor activity data were analyzed by one-way ANOVA followed by Dunnett’s or Tukey’s *post hoc* tests. Fiber-photometry data were analyzed using an unpaired *t* test or two-way ANOVA with Fisher’s LSD post hoc test. RT-qPCR data were analyzed using a bifactorial mixed-effects model with Tukey’s multiple comparisons test. Statistical details are provided in the figure legends. Analyses were performed using GraphPad Prism v10.0 (GraphPad Software, San Diego, CA).

## Results

### Effect of BID on D_1_R agonist-induced locomotor activation

**Fig. 1:**
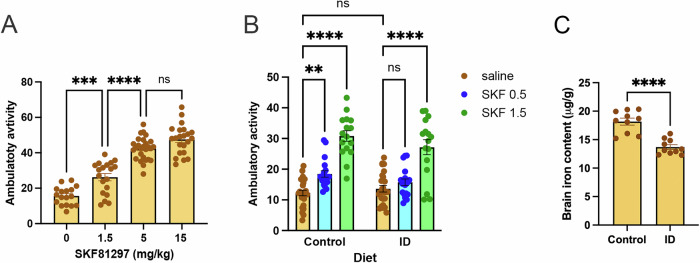
Locomotor activating effect of the systemic administration of the D_1_R agonist SKF81297 in mice with normal and iron-deficient diet. **A** Dose response of SKF81297 (SKF; 0, 1.5, 5 and 15 mg/kg, i.p.) in mice with control diet. **B** Effect of SKF (0.5 or 1.5 mg/kg, i.p.) or vehicle (saline) in mice with control and iron-deficient (ID) diets. Values are means ± S.E.M. and are expressed as the average of the transformed counts (squared root) obtained from the 10 min-periods recorded for 40 min after the systemic administration of SKF or saline. **C** Brain iron content measured in dissected dorsal striatum samples from male and female mice (1:1 ratio) maintained on control or ID diets. In (**A**), one-way ANOVA followed by Dunnett’s post hoc comparisons: ***p < 0.001; ****p < 0.0001; ns non-significant; *n* = 11–19. In (**B**), two-way ANOVA followed by Tukey’s post hoc comparisons: iron effect and SKF effect, *p* = 0.134 and *p* < 0.0001, respectively; **p < 0.01; ****p < 0.0001; ns non-significant; *n* = 16–25. In (**C**), Student’s non-paired *t* test: ****p < 0.0001, *n* = 10/group.

Locomotor activity experiments were first conducted to determine the systemic (i.p.) doses of the D_1_R agonist SKF for subsequent EPN GABA release studies. In control-diet mice, dose–response analysis showed that 1.5 mg/kg produced a minimal but significant increase in locomotion, whereas 5 and 15 mg/kg elicited maximal responses (Fig. 1A). Accordingly, doses of SKF ≤ 1.5 mg/kg and ≥5 mg/kg are referred to as “low” and “maximal,” respectively. Nonlinear regression analysis yielded an EC_50_ of 2.5 mg/kg (GraphPad Software). A second set of experiments compared the effects of low SKF doses (0.5 and 1.5 mg/kg) in mice fed control or ID diets. ID-diet mice appeared less sensitive to SKF, as indicated by a reduced response to 0.5 mg/kg. However, both groups showed similar locomotor activation at 1.5 mg/kg (Fig. 1B). These results suggest that BID does not enhance D_1_R-mediated locomotor mechanisms and may instead attenuate them. The effectiveness of the ID diet in inducing BID was confirmed by measuring brain iron content in randomly selected control- and ID-diet mice (Fig. 1C). Results were comparable to those previously reported using the same diet in this and additional mouse strains [28].

### Effect of BID on D_1_R agonist-induced EPN GABA release

**Fig. 2:**
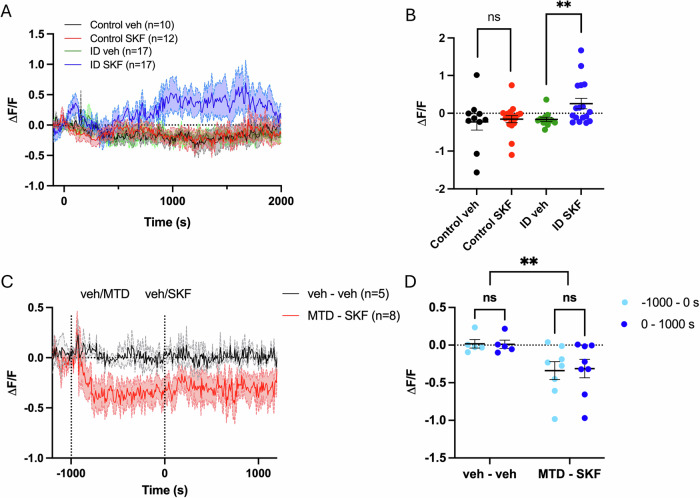
Effect of iron-deficient diet on GABA release in the EPN induced by the systemic administration of a low dose of the D_1_R agonist SKF81297 and methadone. **A** Plots show the time course (seconds, s) of the mean fiber-photometry signal (mean ± S.E.M.) in mice fed an iron-deficient (ID) or control diet following systemic administration of SKF81297 (SKF; 1.5 mg/kg, i.p.; *n* = 17 for ID and *n* = 12 for control) or vehicle (veh; *n* = 17 for ID and *n* = 10 for control). **B** Statistical comparisons of the results shown in (**A**), shown as individual values and means ± S.E.M. of the data from 0 to 2000 s after SKF administration of the four different groups. One-way ANOVA followed by Tukey’s post hoc comparisons: **p < 0.01; ns non-significant. **C** Plots show the time course (s) of the mean fiber-photometry signal (mean ± S.E.M.) in mice with ID diet after systemic administration of methadone (MTD; 10 mg/kg, s.c.) followed 1000 sec after (at time 0) by a subsequent administration of SKF (1. 5 mg/kg, i.p.) (*n* = 8), and a control group with two corresponding vehicle administrations (*n* = 5). **D** Statistical comparisons of the results shown in (**C**), shown as individual values and means ± S.E.M. of the data from −1000 to 0 sec and 0 to 1000 sec of both groups. Two-way ANOVA followed by Fisher’s LSD post-hoc comparisons: **p < 0.01; ns non-significant.

We next measured SKF-induced GABA release in the EPN of control- and ID-diet mice and assessed the effect of methadone. GABA release in the EPN triggered by a D_1_R agonist is considered to reflect activation of both matrix D_1_R neurons and striosomal MOR–D_1_R neurons, the latter being selectively modulated by opioids. To validate the fiber-photometry approach, we first examined the effect of systemic administration of the GABA_A_ receptor agonist muscimol (1 mg/kg, i.p.) in control-diet mice expressing the muscimol-sensitive GABA biosensor iGABASnFR in the EPN (Supplementary Fig. S1A). Muscimol significantly increased fluorescence compared with vehicle (Supplementary Fig. S1B). A maximal locomotor-activating dose of SKF (10 mg/kg, i.p.) likewise produced a significant increase in EPN GABA release relative to vehicle (Supplementary Fig. S1C). To assess potential D_1_R hypersensitivity under BID conditions, we administered a low locomotor-activating dose of SKF (1.5 mg/kg, i.p.). This dose did not alter GABA release in control-diet mice but induced a significant increase in ID-diet mice (Fig. 2A, B). Finally, in ID-diet mice, methadone (10 mg/kg, s.c.), a dose previously shown to produce robust locomotor activation [29], significantly reduced spontaneous EPN GABA release and completely prevented the SKF-induced increase when given 1000 s (16.7 min) prior to SKF (Fig. 2C, D). This antagonistic effect suggests that the enhanced EPN GABA release elicited by low-dose SKF in ID-diet mice is driven by activation of striosomal MOR–D_1_R neurons, or at minimum that the ID diet preferentially increases the sensitivity of these neurons relative to matrix D_1_R neurons.

### Effect of a MOR agonist on D_1_R agonist-induced locomotor activation

**Fig. 3:**
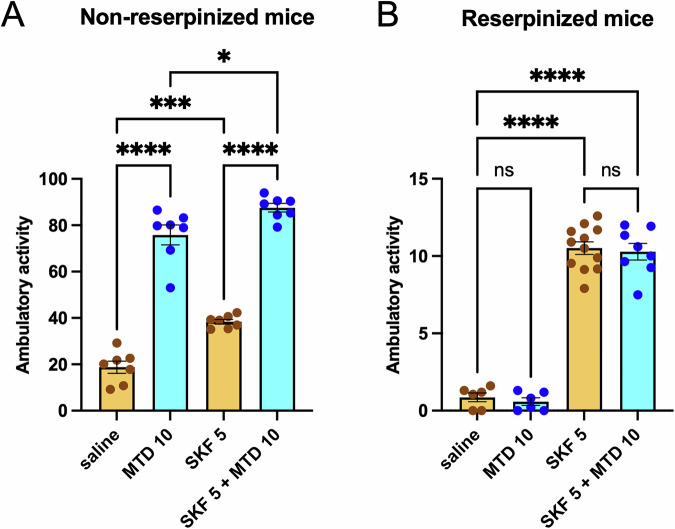
Locomotor-activating effect of the combined systemic administration of the D_1_R agonist SKF81297 and methadone in non-reserpinized and reserpinized mice. In (**B**) mice were administered reserpine 20 h before administration of SKF81297 (5 mg/kg, i.p.; SKF 5) or vehicle (saline). In (**A**) and (**B**) methadone (10 mg/kg, s.c.; MTD 10) or vehicle were administered 15 min before SKF. Values are means ±S.E.M. and are expressed as the average of the transformed counts (squared root) obtained from the 10 min-periods recorded for 40 min after the systemic (i.p.) administration of SKF or saline. One-way ANOVA followed by Tukey’s post hoc comparisons: *p < 0.05; ***p < 0.001; ****p < 0.0001; ns non-significant difference; *n* = 6–12.

Methadone’s inhibition of EPN GABA release contrasts with its locomotor-activating effects, which have been attributed to MOR-dependent increases in mesencephalic dopamine release [29]. Because both methadone and SKF stimulate locomotion, this dissociation suggests that striosomal MOR–D_1_R neurons regulating EPN GABA release are not primary mediators of D_1_R agonist-induced locomotor activation. Rather, SKF-induced locomotion is likely driven predominantly by matrix D_1_R neurons, which do not express MOR. Under this scenario, methadone would not be expected to antagonize SKF-induced locomotor activity. We tested this hypothesis in both naïve and reserpinized mice. Reserpinized mice constitute a classic model of dopamine depletion, enabling assessment of postsynaptic striatal responses to dopamine receptor agonists without interference from endogenous dopamine [19, 22, 24]. This model therefore allows evaluation of a potential modulatory role of postsynaptic MORs in D_1_R-mediated locomotor activity, independent of MOR-driven dopamine release. In non-reserpinized mice, coadministration of maximally effective locomotor-activating doses of SKF (5 mg/kg, i.p.; present study) and methadone (10 mg/kg, s.c. [29]) produced an additive effect, with greater locomotor activation than either drug alone (Fig. 3A). In reserpinized mice, SKF (5 mg/kg, i.p.) still induced locomotion, although to a lesser extent than in non-reserpinized animals (Fig. 3B). Notably, the robust locomotor effect of methadone observed in non-reserpinized mice was completely abolished in reserpinized animals, and methadone did not modify the response to SKF (Fig. 3B). Together, these findings strongly support the conclusion that D_1_R agonist-induced locomotor activation results primarily from direct stimulation of postsynaptic D_1_Rs in matrix neurons, whereas striosomal MOR–D_1_R neurons do not significantly contribute to this behavioral effect.

### Effect of BID on the striatal expression of *Drd1*, *Adora1* and *Oprm1*

**Fig. 4:**
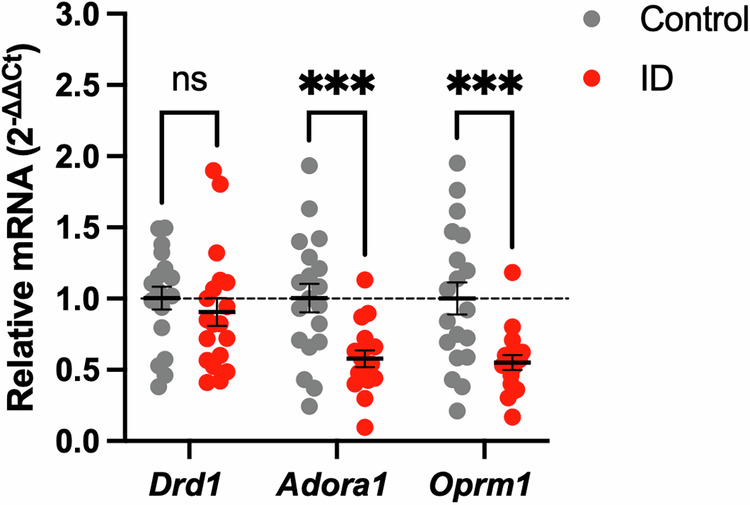
Effect of iron-deficient diet on the striatal expression of *Drd1*, *Adora1* and *Oprm1.* Results represent the striatal receptor mRNA expression of genes encoding D_1_Rs, A_1_Rs, and MORs (*Drd1*, *Adora1*, and *Oprm1*) in mice with control and iron-deficient (ID) diet and is expressed as means ±S.E.M (*n* = 17–9) of the relative mRNA expression, normalized as 1.0 for the control group. Bifactorial mixed-effects model followed by Tukey’s multiple comparisons test: ***p < 0.001; ns non-significant difference.

Adenosine A_1_ receptors (A_1_Rs) exert inhibitory control over striatal D_1_R function through receptor heteromerization [19–21]. Similarly, MORs form heteromers with D_1_Rs in the striatum and negatively modulate D_1_R signaling [30, 31]. In previous studies, we reported a significant reduction in striatal A_1_R density in rodents with BID [32], a change that could enhance D_1_R sensitivity and represent a potential pathogenic mechanism in RLS [33, 34]. A BID-induced decrease in striatal MOR density could produce a similar effect. To address this possibility, we compared striatal mRNA expression of genes encoding D_1_Rs, A_1_Rs, and MORs (*Drd1*, *Adora1*, and *Oprm1*) in mice fed control or ID diets using qPCR. The striatal mRNA expression of the housekeeping gene *GAPDH* was not significantly modified with the ID diet (Supplementary Fig. S2). ID-diet mice showed significant reductions in both A_1_R and MOR expression (Fig. 4), whereas D_1_R expression remained unchanged (Fig. 4).

## Discussion

The contrasting effects of MOR activation and BID on D_1_R-agonist-induced locomotor responses and EPN GABA release likely arise from their distinct influences on two neuronal populations: opioid-insensitive matrix D_1_R neurons and opioid-sensitive striosomal MOR-D_1_R neurons. The results indicate that D_1_R agonist-induced locomotor activity is mostly mediated by the matrix-D_1_R-neurons, and that BID selectively increases the sensitivity of the striosomal MOR-D_1_R neurons to the EPN GABA releasing effects of D_1_R agonists.

**Fig. 5:**
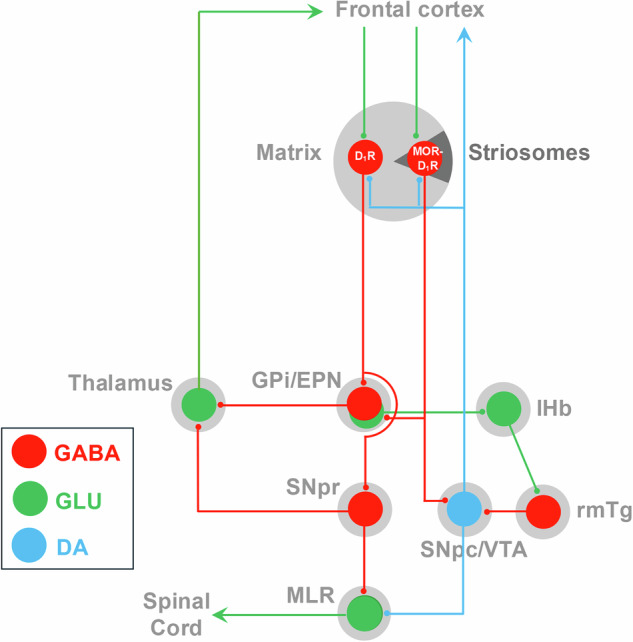
Scheme of the outputs of the striatal matrix D_1_R and striosomal MOR-D_1_R neurons. The striosomal compartment comprises approximately 15% of the total striatal volume and contains GABAergic projection neurons that co-express D_1_Rs and MORs. It has been proposed that a selective increase in dopaminergic sensitivity of these striosomal MOR–D_1_R neurons may represent a key pathogenic mechanism underlying the akathisia observed in RLS and the restlessness associated with opioid withdrawal. Abbreviations: iGP/EPN, internal segment of the globus pallidus, entopeduncular nucleus; lHb, lateral habenula; MLR, mesencephalic locomotor region; rmTg, rostromedial tegmental nucleus; SNpc, substantia nigra pars compacta; SNpr, substantia nigra pars reticulata; VTA, ventral tegmental area. For simplification, only the cortex is shown as an input to the striatum, but it also receives inputs from the midline and intralaminar thalamic nuclei, and the ventral striatum also receives inputs from the hippocampus and amygdala.

The matrix D_1_R-neurons project to GABAergic neurons of the SNpr which project to glutamatergic neurons of the thalamus, and to the mesencephalic locomotor region (MLR), while the striosomal MOR-D_1_R neurons project to the dopaminergic neurons of the substantia nigra pars compacta (SNpc) and ventral tegmental area (VTA) (Fig. 5) [16, 35–38]. Activation of the matrix D_1_R-neurons promotes locomotor activation by disinhibition of the glutamatergic cells of the MLR [36, 37]. On the other hand, recent studies suggest that the selective activation of striosomal MOR-D_1_R-neurons can inhibit motor activity by their direct inhibitory connection with the dopaminergic cells of the SNpc and VTA [38, 39]. This inhibition of dopaminergic cell function should then result in decreased locomotor activity due to the decrease in striatal dopamine release and the concomitant decrease in the activation of postsynaptic striatal dopamine receptors, but also by a decrease in dopamine release in the MLR mediated by a direct innervation from the mesencephalic dopaminergic cells [40] (Fig. 5). The lack of methadone-induced inhibition of D_1_R agonist-induced locomotor activity in reserpinized mice, which is independent of endogenous dopamine, indicates that D_1_R agonist-induced locomotor activation is mostly mediated by the matrix-D_1_R-neurons. The same mechanism would apply to the D_1_R agonist-induced locomotor activation in non-reserpinized mice, indicating a final predominant locomotor-activating effect of the matrix-D_1_R-neurons over the inhibitory effect of the striosomal MOR-D_1_R-neurons that project to the dopaminergic cells.

To better understand the predominant excitatory contribution of matrix D_1_R neurons, as opposed to the potential inhibitory influence of striosomal MOR-D_1_R neurons on the locomotor effects of D_1_R agonists, it is important to consider the anatomical organization of the striatum. Striosomes account for ~15% of the total striatal volume (Fig. 5) and are particularly prominent in the anterior and medial regions of the striatum. Interestingly, they predominate in the medial shell of the nucleus accumbens, a region enriched in MORs and D_1_R-expressing neurons that project monosynaptically to dopaminergic neurons in the VTA [41–43]. Opioid-mediated inhibition of these inhibitory striosomal MOR-D_1_R neurons may underlie the methadone-induced potentiation of SKF-evoked locomotor activation in non-reserpinized animals. Conversely, the reduced D_1_R agonist-induced locomotor activation observed in mice fed an ID diet may reflect increased sensitivity of these inhibitory striosomal MOR-D_1_R neurons.

Additionally, both the matrix D_1_R-neurons and the striosomal MOR-D_1_R-neurons also project to the GPi/EPN, but to different functional neuronal populations. The matrix D_1_R-neurons project to GABAergic neurons of GPi/EPN which project to the thalamus, while striosomal MOR-D_1_R-neurons project to GPi/EPN neurons that co-express GABA and glutamate and project to the lHb [14, 18, 44, 45] (Fig. 5). Our results indicate that BID specifically increases the sensitivity of the striosomal MOR-D_1_R neurons to the EPN GABA releasing effects of D_1_R agonists. In a previous study in mice [14], MOR agonists were found to exert a strong inhibition of the ability of striosomal MOR-D_1_R-neurons to release GABA in the EPN and specifically onto the EPN neurons that project to the lHb neurons. In agreement, here we found that methadone significantly decreased basal and SKF-induced GABA release in the EPN. Yet, the same dose of methadone did not modify the locomotor activation induced by SKF in reserpinized mice, agreeing with the lack of involvement of the striosomal MOR-D_1_R-neurons in the locomotor activation induced by D_1_R activation. Furthermore, although matrix D_1_R neurons also project to the EPN, they did not contribute to the BID-induced sensitization of D_1_R-agonist-evoked GABA release in this nucleus. Otherwise, we would expect an enhanced locomotor response to the D_1_R agonist; instead, a reduced response was observed

The lHb is a hub of the striatal striosomal evaluation circuitry running in parallel with the striatal matrix selection circuitry through the two functionally separated “non-motor” and “motor” components of the GPi/EPN [18, 45–48]. Activation of the lHb leads to aversion and motor arrest by virtue of its significant indirect connection to SNpc/VTA dopaminergic neurons through the rostromedial tegmental nucleus (rmTG) (Fig. 1) [49, 50]. The tonic activity of the GPi/EPN-lHb neurons leads to a net excitatory effect of the lHb which is counteracted by the intermittent GPi/EPN GABA release from the striosomal MOR-D_1_R-neurons [14, 50–52]. Therefore, activation of the striosomal MOR-D_1_R-neurons that inhibit the GPi/EPN-lHb pathway promotes disinhibition of the dopaminergic cells, balancing the opposing effect of the directly projecting striosomal MOR-D_1_R-neurons. But the lHb is not just a relay station in the striatal striosomal evaluation circuitry, as simplified in Fig. 5, and integrates additional direct inputs from other brain areas, especially from the medial prefrontal cortex and hypothalamus [50, 53].

Since, as mentioned in the Introduction, dietary-induced BID in mice constitutes a rodent model of RLS with significant construct validity [4, 54], the present results imply that a selective increased dopaminergic sensitivity of the opioid-responsive striatal-EPN-lHb pathway might represent a pivotal pathogenetic mechanism involved in the akathisia of RLS and the restlessness of opioid withdrawal. Furthermore, the results support the hypothesis that the striosomal MOR-D_1_R neurons represent a main target involved in the therapeutic effect of opioids in RLS. It could be argued that the evidence implicating striosomal MOR–D_1_R neurons is largely indirect, since their activity was not directly measured and the study relied on systemic administration of D_1_R and MOR agonists. However, functional D_1_Rs and MORs are present not only in the somatodendritic compartment but also at the EPN terminals of MOR–D_1_R neurons. In vivo microdialysis studies have shown that D_1_R agonists applied directly to either the striatum or the EPN increase GABA release in the EPN [20]. Moreover, a recent study using viral and optogenetic approaches in sagittal mouse brain slices containing the striatum and EPN demonstrated, in selectively identified striosomal MOR–D1R neurons, that direct application of MOR agonists robustly and antagonistically modulates GABA release onto EPN–LHb neurons [14]. Thus, although indirect influences from other brain regions cannot be excluded, functionally significantly D_1_Rs and MORs localized at the EPN terminals of the striosomal MOR–D_1_R neurons likely predominate in determining the net effect of systemically administered ligands on EPN GABA release.

The RLS rodent model with dietary-induced BID has also shown its translational predictive value. Using an in vivo optogenetic-microdialysis approach, we previously found that in the rat with BID there is an increased sensitivity of the corticostriatal neuronal terminals to release glutamate [26]. We could also demonstrate that the α_2_δ ligand gabapentin and the D_2_-like receptor agonists pramipexole and ropinirole target these terminals and inhibit corticostriatal glutamate release, providing a very plausible mechanism for their therapeutic effect in RLS [26]. We then demonstrated that the increased sensitivity of corticostriatal terminals depends on a BID-induced reduction in the expression of inhibitory presynaptic adenosine receptors of the A_1_R subtype localized in those terminals [55, 56]. This led to the prediction of a therapeutic efficacy of inhibitors of adenosine transporters, like dipyridamole, in RLS [57].

Our findings indicate that BID induces an additional postsynaptic striatal alteration, resulting in preferential activation of striosomal MOR-D_1_R neurons. We initially hypothesized that this increased sensitivity was due to BID-induced downregulation of postsynaptic A_1_Rs, which are expressed in D_1_R-positive striatal neurons projecting to the EPN and whose activation inhibits D_1_R agonist-induced EPN GABA release [20]. Consistent with this, qPCR analysis revealed a significant BID-associated reduction in striatal A_1_R mRNA, indicating A_1_R downregulation in striatal neurons beyond corticostriatal terminals. However, this effect appears selective for striosomal MOR-D_1_R neurons, as matrix D_1_R neuron sensitivity was unaffected. Supporting this interpretation, A_1_R agonists attenuate and A_1_R antagonists potentiate D_1_R agonist-induced locomotion in reserpinized mice [19, 58], and BID did not enhance locomotor responses to low-dose D_1_R agonists.

Increased sensitivity of striosomal MOR-D_1_R neurons may additionally involve functional downregulation of MORs. A PET study using [¹¹C]diprenorphine reported negative correlations between opioid receptor binding and RLS severity in the striatum, suggesting either an increased endogenous opioid tone or reduced MOR density [7]. The latter interpretation is supported by the clinical efficacy of MOR agonists and the lack of effect of naloxone in RLS [5, 59]. In agreement, our qPCR data show a marked reduction in striatal MOR expression in BID mice. Ongoing studies are examining BID effects on striatal MOR-D_1_R heteromerization and function.

Experiments were conducted during the inactive period, when only a trend toward increased locomotor activity was observed in iron-deficient mice, consistent with reports that BID-related hyperactivity is circadian dependent and maximal late in the active (dark) phase [60]. Future studies will address this component, particularly given the role of the lHb in circadian regulation downstream of the suprachiasmatic nucleus [61]. Nevertheless, RLS is characterized by an urge to move the legs rather than by increased ambulatory activity per se, and our findings are consistent with a modulatory, rather than direct, role of the striosomal versus matrix components of the striatal–EPN pathway in the regulation of locomotor activity. If a selectively increased sensitivity of the opioid-responsive striatal–EPN pathway represents a key pathogenetic mechanism in RLS, this raises concerns about whether open-field locomotion adequately captures the relevant akathisia-like phenotype and how well the current behavioral readouts reflect the clinical features of RLS. Overall, this study provides new mechanistic insight into akathisia in RLS and opioid withdrawal and introduces a model for evaluating opioids and other therapies targeting cortico-subcortical circuits involving striosomal MOR-D_1_R neurons and the GPi/EPN-lHb pathway.

## Supplementary information

Supplementary Figures

## Data Availability

Data will be made available on request.
