# Supplementary material for: Dopaminergic hypersensitivity of the opioid-responsive striatal–entopeduncular pathway in a rodent model of restless legs syndrome
Source: Neuropsychopharmacology. 2026 Apr 17;51(10):1865–72. doi: 10.1038/s41386-026-02413-2 (PMC13377859; doi:10.1038/s41386-026-02413-2)
Supplement: Supplementary file 1 [file 41386_2026_2413_MOESM1_ESM.pdf]

Supplementary Figures

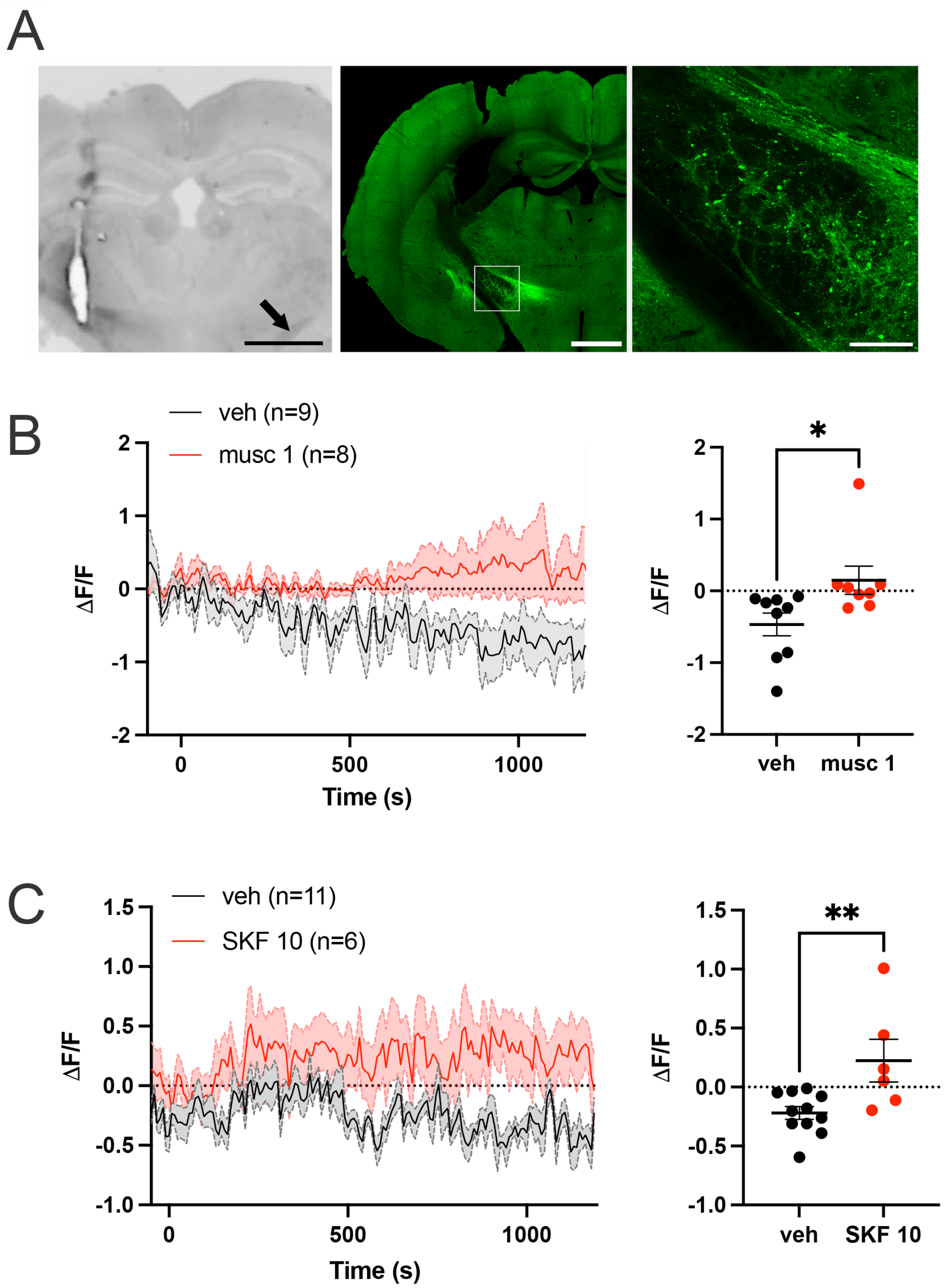

Fig. S1. Activation of the GABA biosensor injected in the EPN upon systemic administration of the $GABA_A$ receptor agonist muscimol or the D_1R agonist SKF81297 (SKF) in mice with control diet. (A) Left picture: coronal brain section of the mouse brain at the level of the EPN (arrow) showing the probe track and placement (bar length: 1 mm); middle picture: coronal brain section of the mouse brain at the level of the EPN (framed area) showing the localization of infected cells expressing the GABA biosensor iGABASnFR [25] (bar length: 1 mm); right picture: amplified framed area from the middle picture (bar length: 0.2 mm). (B) Left graph: Plots show the time course (seconds, s) of the mean fiber-photometry signal (mean ± S.E.M.) in mice fed a control diet

following systemic administration of muscimol (1 mg/kg, i.p.) or vehicle (veh) (n = 8 and 9, respectively); right graph: statistical comparisons of the results shown in the left graph; results are shown as individual values and means ± S.E.M. of the data from 0 to 1200 s after muscimol administration. (C) Left graph: Plots show the time course (s) of the mean fiber-photometry signal (mean ± S.E.M.) in mice fed a control diet after systemic administration of SKF (10 mg/kg, i.p.) or vehicle (veh) (n = 6 and 11, respectively); right graph: statistical comparisons of the results shown in the left graph; results are shown as individual values and means ± S.E.M. of the data from 0 to 1200 s after SKF administration. Non-paired *t* test: *p < 0.05, ***p < 0.01.

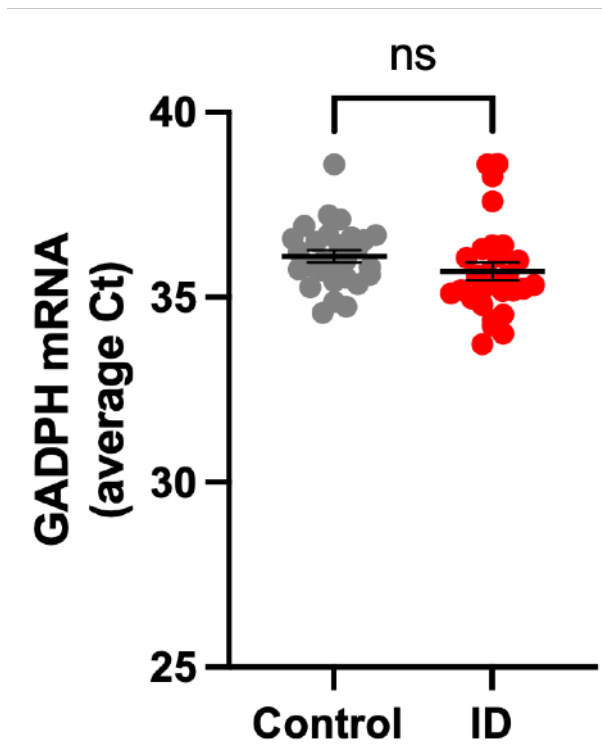

Fig. S2. Lack of effect of iron-deficient diet on the striatal expression of the housekeeping gene *Gapdh*. Results represent the striatal mRNA expression of GAPDH in mice with control and iron-deficient (ID) diet and is expressed as means ± S.E.M (n = 28/group) of Ct values (see Materials and Methods). Unpaired t test: ns, non-significant difference.
